# Supplementary material for: Engineering Bacillus subtilis for the formation of a durable living biocomposite material
Source: Nat Commun. 2021 Dec 8;12:7133. doi: 10.1038/s41467-021-27467-2 (PMC8654922; doi:10.1038/s41467-021-27467-2)
Supplement: Supplementary file 1 — Supplementary Information [file 41467_2021_27467_MOESM1_ESM.pdf]

Engineering *Bacillus subtilis* for the formation of a durable living biocomposite material

Kang *et al.*

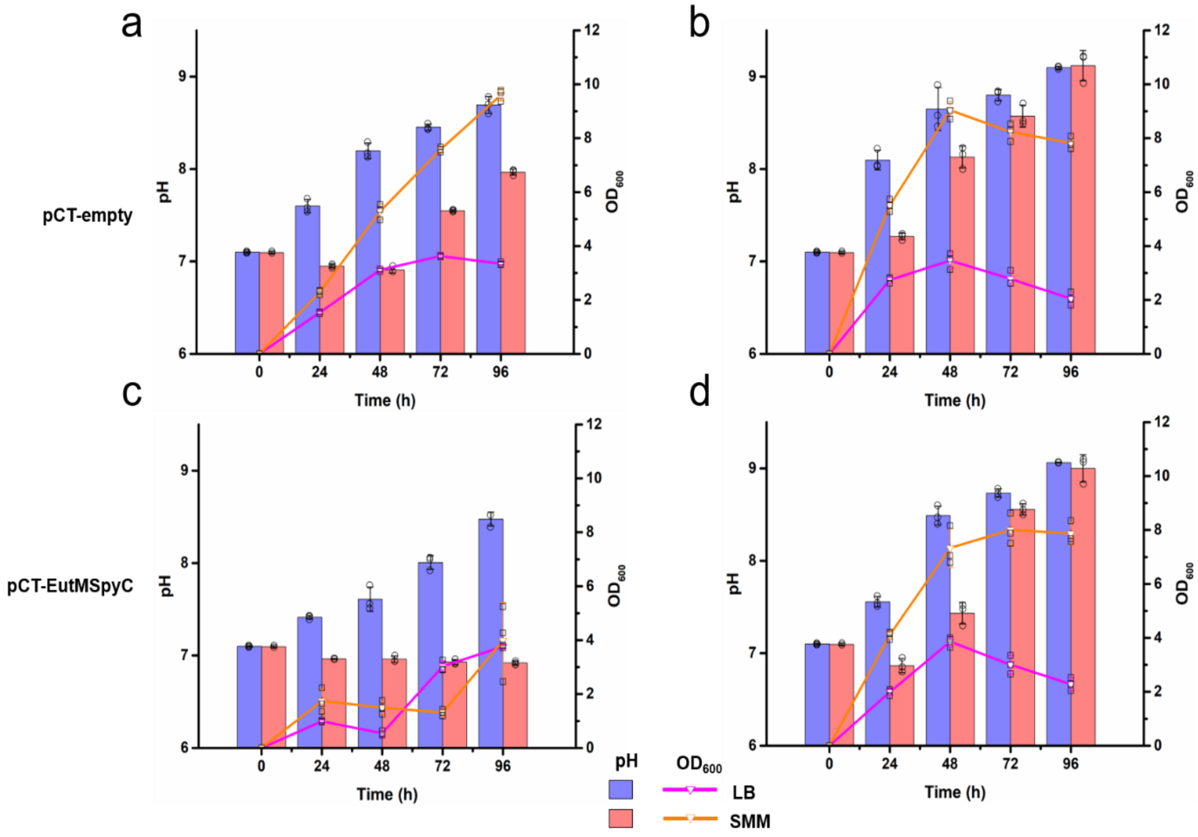

Supplementary Fig. 1. Establishing growth conditions suitable for silica biocomposite formation with *B. subtilis* pCT-empty and pCT-EutMSpyC.

Growth of *B. subtilis* WT strain transformed with the cumate inducible, pCT-empty expression plasmid was compared in two media (LB and SMM, 100 rpm) at (**a**) 20°C and (**b**) 30°C. Similarly, growth of *B. subtilis* WT transformed with pCT-EutMSpyC was compared in LB and SMM at (**c**) 20°C and (**d**) 30°C. OD_{600} and pH of cultures were followed for 96 h. Cultures were induced with 10 µM cumate once they reached an OD_{600} of 0.4-0.7. *B. subtilis* WT strain transformed with pCT-EutMSpyC (**c**) show changes in growth rates at 20°C suggesting diauxic growth and/or an increased metabolic burden caused by recombinant protein expression. Data are shown as mean values ± SD and error bars represent the standard deviations of three independent biological replicate cultures. Colored bars and lines represent mean values. Black symbols represent data points and error bar. Source data are provided as a Source Data file.

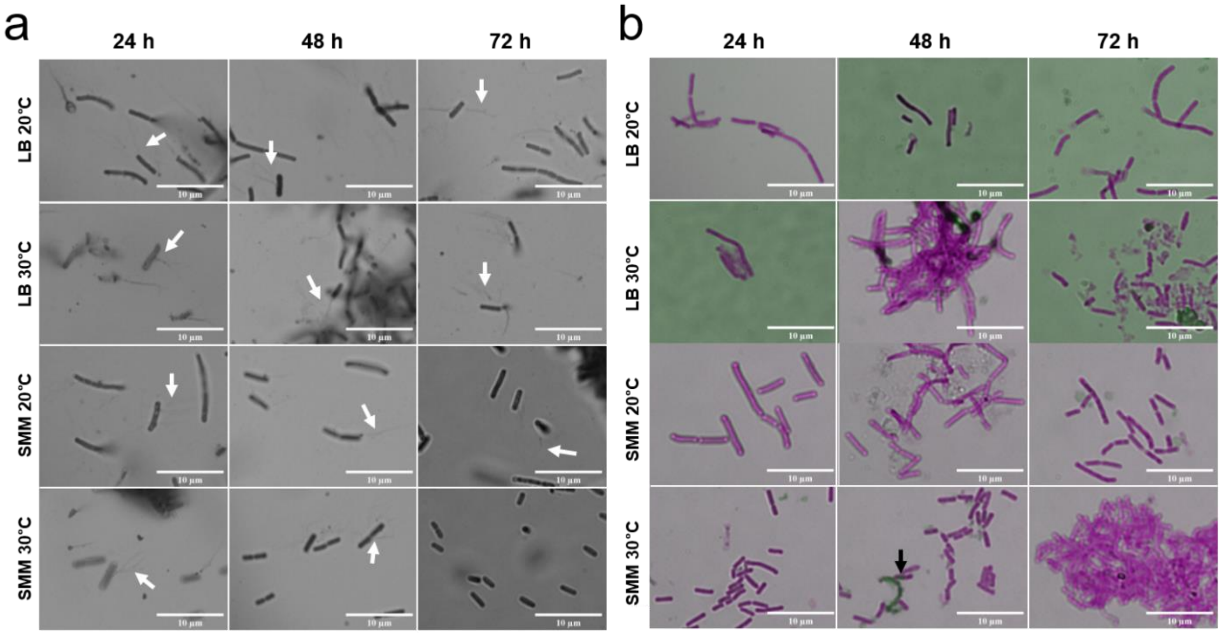

Supplementary Fig. 2. Analysis of spore formation and flagella phenotypes of *B. subtilis* pCT-empty under different growth conditions.

Spore formation and flagellation of *B. subtilis* WT pCT-empty was analyzed under different growth conditions. At 24 h time intervals, samples were removed from SMM and LB cultures grown at 20°C or 30°C (shown in Supplementary Fig. 1) for staining and imaging by light microscopy. Cells were stained with RYU to observe flagella (**a**) or malachite green and safranin red to visualize spores (**b**). Flagella were observed through 72 h of growth, with the most flagella present at 48 h of growth or less under all conditions. Only one, unreleased spore was observed at 48 h of growth in SMM at 30°C, corresponding to reaching stationary phase. The images are representative of three independent biological replicate cultures.

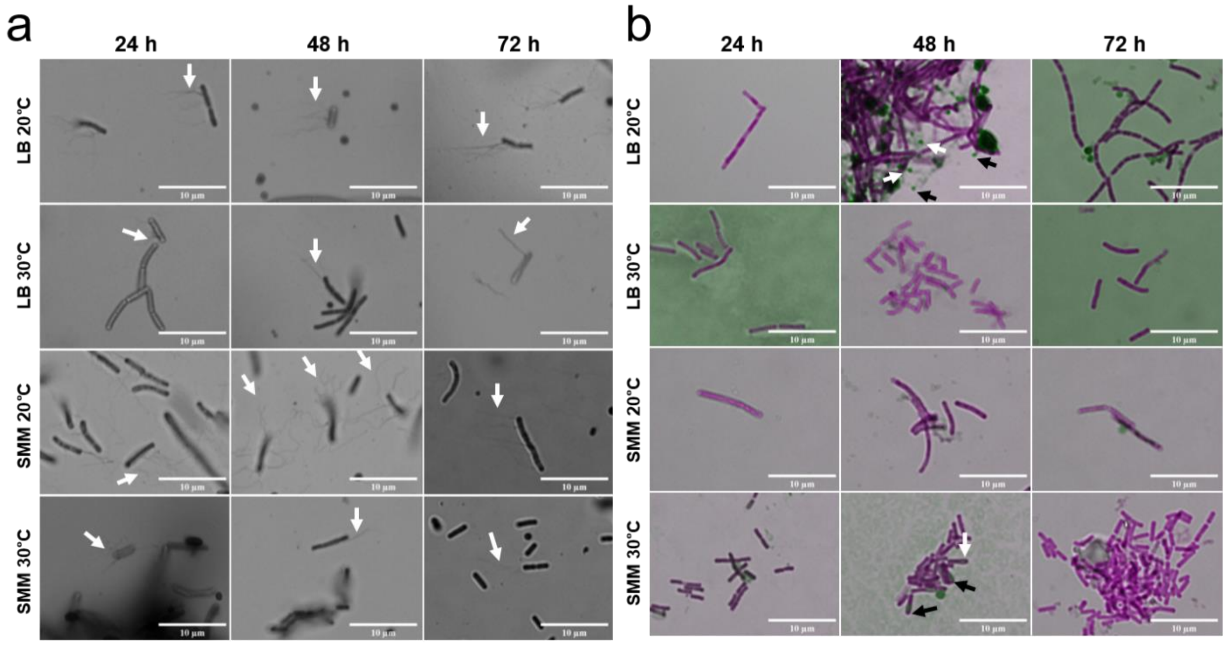

Supplementary Fig. 3. Analysis of spore formation and flagella phenotypes of *B. subtilis* pCT-EutMSpyC.

Spore formation and flagellation of *B. subtilis* WT transformed with pCT-EutMSpyC was analyzed under different growth conditions. At 24 h time intervals, samples were removed from SMM and LB cultures grown at 20°C or 30°C (shown in Supplementary Fig. 1) for staining and imaging by light microscopy. Cells were stained with RYU to observe flagella (**a**) or malachite green and safranin red to visualize spores (**b**). Flagella were observed through 72 h of growth, with the most flagella present at 48 h of growth or less under all conditions. Sporulation was observed after 48 h in LB medium at 20°C, correlating with a corresponding decrease in OD_{600} due to cell lysis (see Supplementary Fig. 1) while the few spores visible at 48 h in SMM at 30°C did not impact OD. (Images are representative of three independent biological replicate cultures.)

a

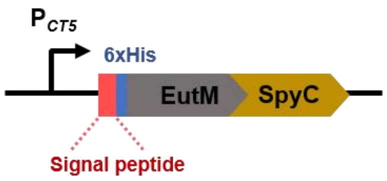

Signal peptide	Amino acid sequence
SacB	MNIKKFAKQATVLTFTTALLAGGATQAFAAST
XynA	MFKFKKNFLVGLSAALMSISLFSATASAAST
YngK	MYIKKCIGSILFLLLFCSSALPAKAAST
CelA	MKRSISIFITCLLITLLTMGGMIASPASAAST
LipA	MKKVLMAFIICLSLILSVLAAPPSGAKAAST

b

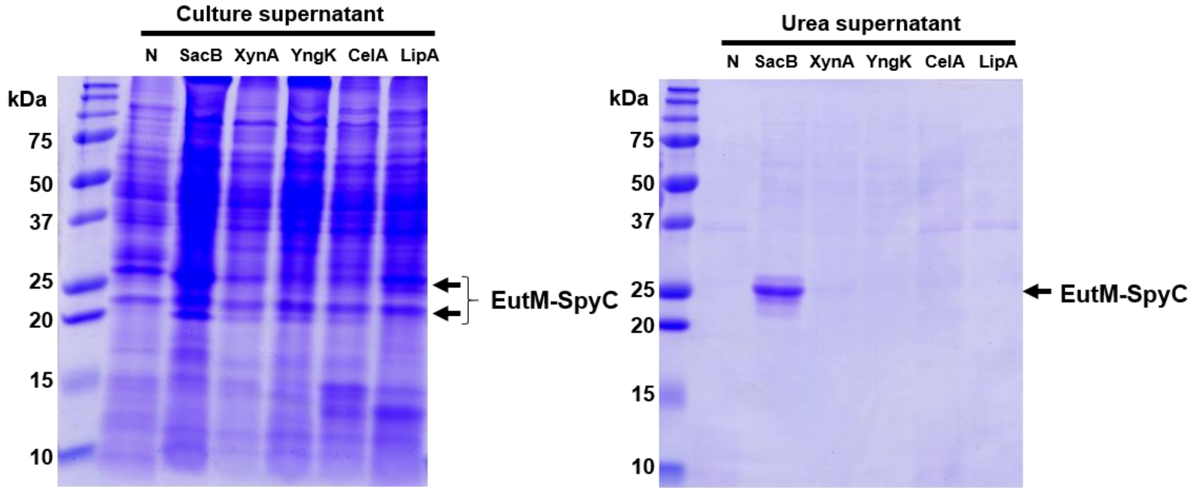

Supplementary Fig. 4. Testing of additional secretion signal sequence for scaffold building block secretion by B. subtilis.

a Scaffold building block secretion levels by *B. subtilis* WT was compared with five commonly used secretion signal peptides placed upstream of the His-EutM-SpyCatcher region.

b Cultures expressing the EutM-SpyCatcher proteins were grown for 48 h in SMM at 20°C under induction condition to analyze protein secretion by SDS-PAGE. Culture supernatants were collected and concentrated 25-fold by TCA precipitation prior to analysis (left panel). Cell pellets were then treated with 4 M urea (see Fig. 2) to solubilize co-precipitated EutM-SpyCatcher scaffolds for analysis (right panel). *B. subtilis* transformed with a pCT-empty vector was included as negative control (N). Only the SacB signal sequence facilitated secretion of EutM-SpyCatcher in the supernatant and as co-precipitant in the cell pellet. SDS-PAGE analysis is representative of three independent biological replicate cultures. Source data are provided as a Source Data file.

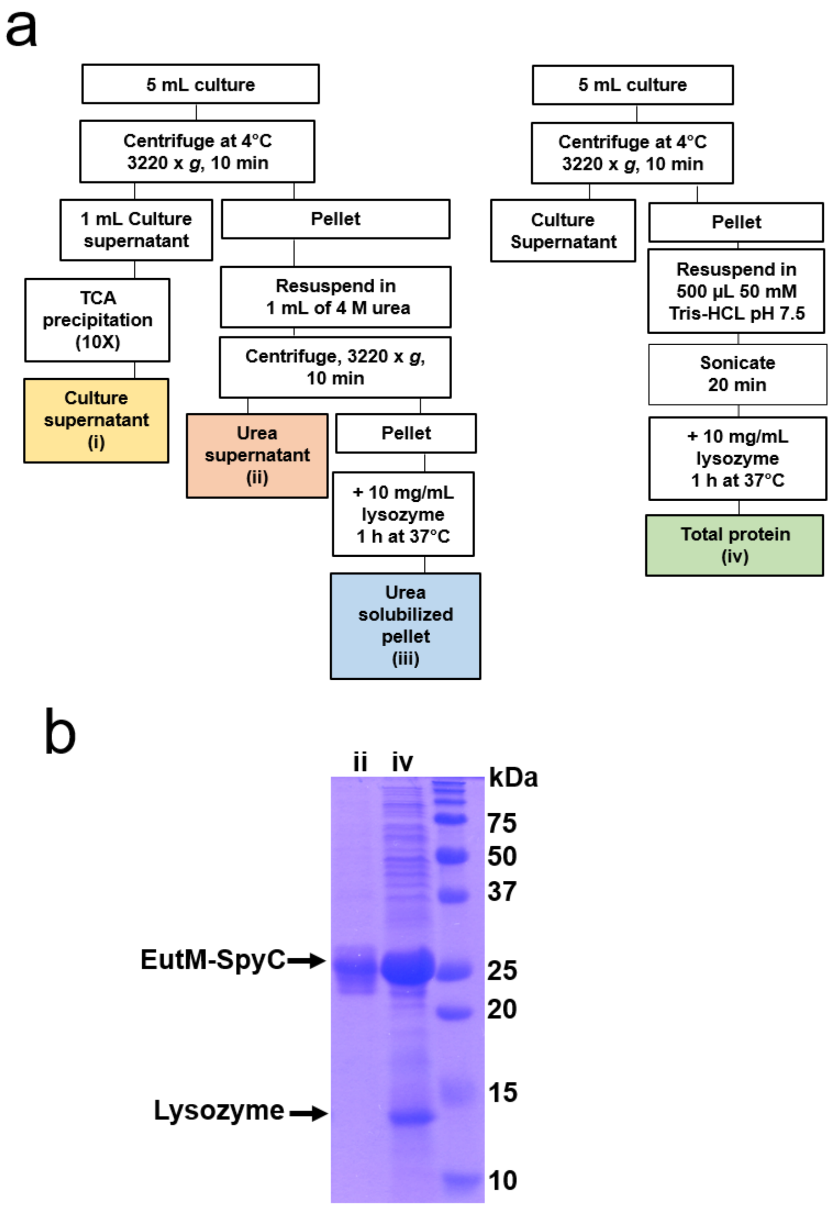

Supplementary Fig. 5. Experimental workflow used for the analysis of EutM scaffold building block secretion and expression by engineered *B. subtilis*.

Different fractions of proteins were prepared by *B. subtilis* WT pCT-EutMSpyC after 48 h of induction in SMM at 20°C.

a Workflow for the preparation of the following fractions containing EutM-SpyCatcher protein: (i) culture supernatant, (ii) urea supernatant, (iii) urea solubilized pellet and (iv) total protein. Fractions i-iii correspond to A, B and C in Fig. 2, 3 & 8. Fraction iv represents total protein (intracellular and precipitated scaffolds) in sedimented pellet after complete lysis of cells.

b Comparison of proteins solubilized by urea treatment from pelleted cells and scaffolds (fraction ii) and total protein released from broken cells and sedimented scaffolds (fraction iv). Urea treatment does solubilize EutM scaffolds but does not break cells. SDS-PAGE analysis is representative of three independent experiments. Source data are provided as a Source Data file.

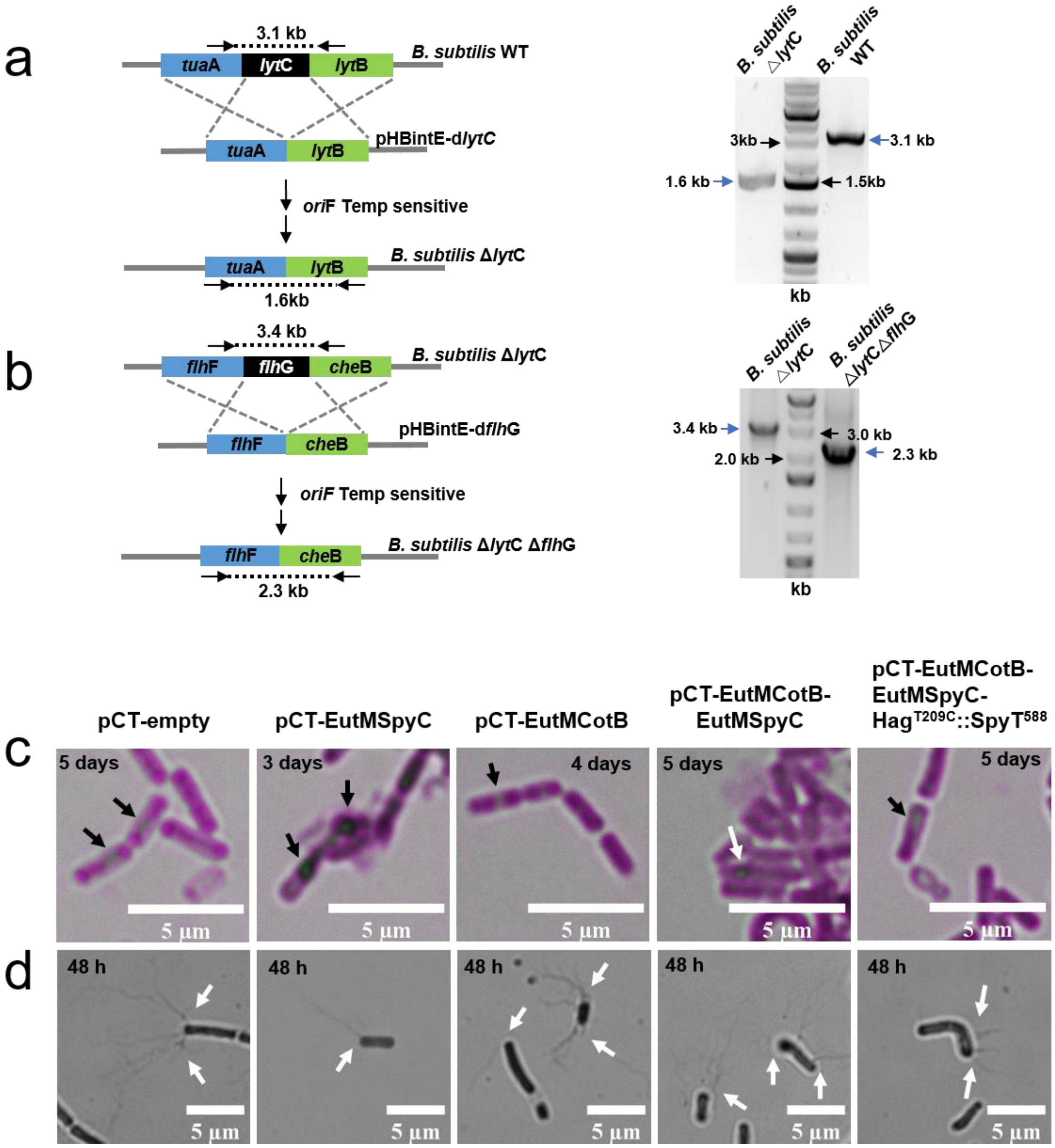

Supplementary Fig. 6. Construction and phenotypical characterization of the double deletion strain *B. subtilis* Δ*lyt*C Δ*flh*G as ELM fabrication chassis.

Deletions of genes encoding the autolysin LytC (**a**) and flagellar basal body protein FlhG (**b**) were made in *B. subtilis* 168 (WT strain) by consecutive rounds of homologous recombination with a temperature sensitive plasmid. In frame deletions were confirmed by PCR, yielding amplification products with the expected sizes. Endospore formation (**c**) and polar flagella clusters (**d**) were then confirmed for the double deletion strain transformed with different expression plasmids used in this work. *B. subtilis* Δ*lyt*C Δ*flh*G strains were cultured in SMM at 20°C under induction condition. Spores were visualized with malachite green and safranin red at days after induction as indicated (when endospores formed). Red was replaced with magenta using FIJI software (**c**). Flagella of the same cultures RYU stained and imaged after 48 h of induction. Images were converted to grayscale using GIMP software (**d**). PCR results in **a**, **b** are from a single experiment and images in **c**, **d** are representative of three independent biological replicate cultures. Source data are provided as a Source Data file.

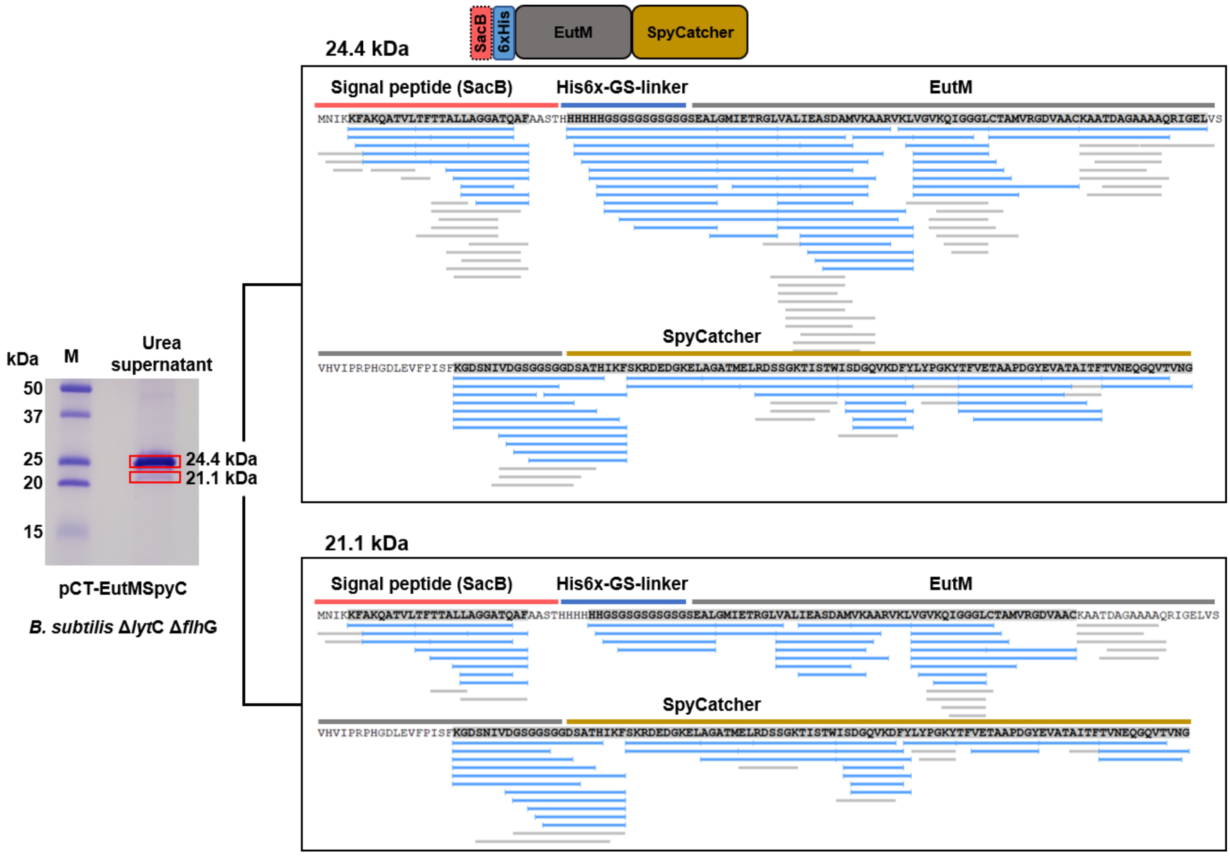

Supplementary Fig. 7. Analysis of EutM-SpyCatcher secreted by *B. subtilis* Δ*lytC* Δ*flhG*.

SDS-PAGE analysis and LC-MS *de novo* sequencing of EutM-SpyCatcher proteins produced by *B. subtilis* Δ*lyt*C Δ*flh*G pCT-EutMSpyC after 48 h of induction in SMM at 20°C. Two bands (21.1 and 24.4 kDa) are visible in the urea supernatant. Boxed bands were cut and subjected to *de novo* sequencing by LC-MS. Coverages of sequenced peptides were visualized by PEAKS Studio Viewer shown to the right. Under these expression conditions, both protein bands have an uncleaved SacB signal peptide. A segment after the signal peptide cleavage site (AFA) was not sequenced in addition to other sequence regions. To confirm that the LC-MS sequencing results were not due to plasmid mutations that occurred during cultivation in the signal peptidase cleavage site regions, plasmids isolated from 10 colonies obtained from the *B. subtilis* Δ*lyt*C Δ*flh*G pCT-EutMSpyC were Sanger sequenced to cover the signal peptide and the EutM-SpyCatcher region. No mutations were found. Peptide fragments sequenced corresponding to the C-terminus of both protein bands indicate that the minor, smaller 21.1 kDa band corresponds to an isoform that migrates faster in the SDS-PAGE gel. SDS-PAGE analysis is representative of three independent experiments (see Fig. 3d). Source data are provided as a Source Data file.

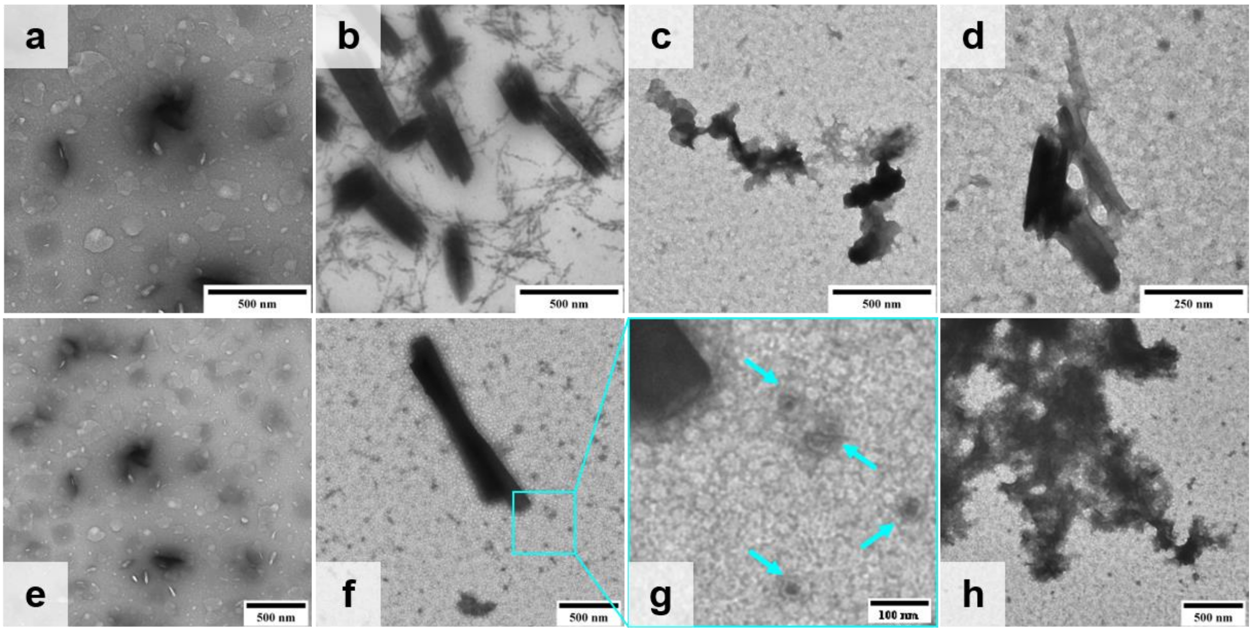

Supplementary Fig. 8. Comparison of scaffold formation by EutM-SpyCatcher purified from recombinant *E. coli* and *B. subtilis* Δ*lyt*C Δ*flh*G cultures.

His-tagged EutM-SpyCatcher from *E. coli* and urea solubilized scaffolds of *B. subtilis* Δ*lyt*C Δ*flh*G pCT-EutMSpyC cultures were purified by metal affinity chromatography as described in the methods. The eluted proteins were dialyzed into SMM (**a**, **e** media blank used for *E. coli* and *B. subtilis* protein dialysis, respectively) to remove imidazole and urea for analysis of scaffold formation in this medium. The SMM dialyzed EutM-SpyCatcher proteins (*E. coli* 1.8 mg/mL, *B. subtilis* 2.3 mg/mL) were diluted to 1 mg/mL with sterile SMM and structures were visualized by TEM. **a-c** are magnified 10,000 x, **d** is 20,000 x, **e**, **f**, **h** are 6,000 x. **g** is enlarged 5x from **f**.
b Rolled up tubes were visible in *E. coli* protein samples and have been observed previously for EutM scaffolds[2,3]. **c, d, f-h** Rolled up tubes, sheets, coral- and small spherical structures (that stain as donut-like structures) were visible in *Bacillus* samples and have also been previously observed for EutM scaffolds [2,3]. The enlarged spherical structures from **f** are also present in **c**, **d**, and **h**. All images are representative of one set of purified proteins.

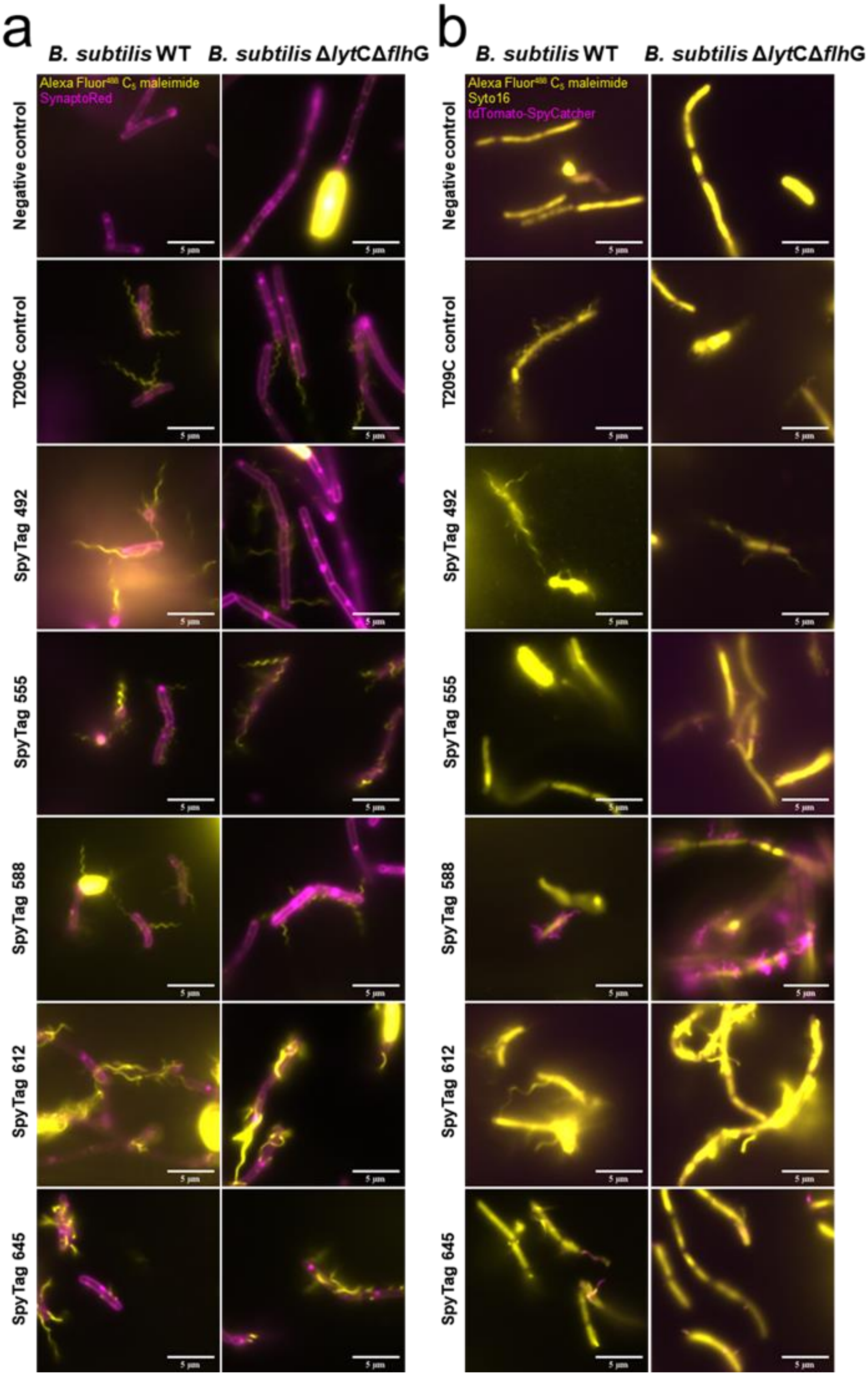
a
B. subtilis WT
B. subtilis ΔlytCΔflhG
Alexa Fluor488 C5 maleimide
SynaptoRed
Negative control
T209C control
SpyTag 492
SpyTag 555
SpyTag 588
SpyTag 612
SpyTag 645
5 µm
b
B. subtilis WT
B. subtilis ΔlytCΔflhG
Alexa Fluor488 C5 maleimide
Syto16
tdTomato-SpyCatcher

Supplementary Fig. 9. Characterization of phenotypes and functions of SpyTag-modified flagella.

B. subtilis WT and *B. subtilis* Δ*lytC* Δ*flh*G strains were transformed with pRBBm34-Hag^{T209C}::SpyT$^{###}$ (dye reactive Cys, SpyTag insertions at indicated nucleotide locations) plasmids and flagella phenotypes and functions compared to strains transformed with pRBBm34-Hag^{T209C} and pCT-empty plasmid as positive and negative controls, respectively.

a Fluorescence microscopy to evaluate flagella morphologies due to SpyTag insertion. Individual colonies from plates were inoculated in 4 mL LB medium (supplemented with tetracycline) and grown overnight at 30°C and 220 rpm. The *hag* gene is expressed from its native promoter. Flagella were stained with Alexa Fluor^{TM} 488 C_5 maleimide and cell membranes with SynaptoRed^{TM} C2. Samples were examined using a Nikon Eclipse 90i microscope at 100X. dsRed (excitation 530-560 nm, emission 590-650 nm) and GFP (excitation 450-490 nm, emission 500-550 nm) channels were captured in black and white. Channels were false colored magenta for RFP and yellow for GFP and merged using FIJI.

b Fluorescence microscopy to evaluate SpyTag function when inserted at different locations of the Hag protein. Cultures were grown as above but with the addition of 0.15 mg purified tdTomato-SpyCatcher protein to label functionally displayed SpyTags on flagella. Flagella were stained with Alexa Fluor^{TM} 488 C_5 maleimide and cells visualized with the nucleic acid stain Syto^{TM} 16. Samples were imaged and channels false colored as above.

Images generated for each strain are representative of cultures from three biological replicate cultures.

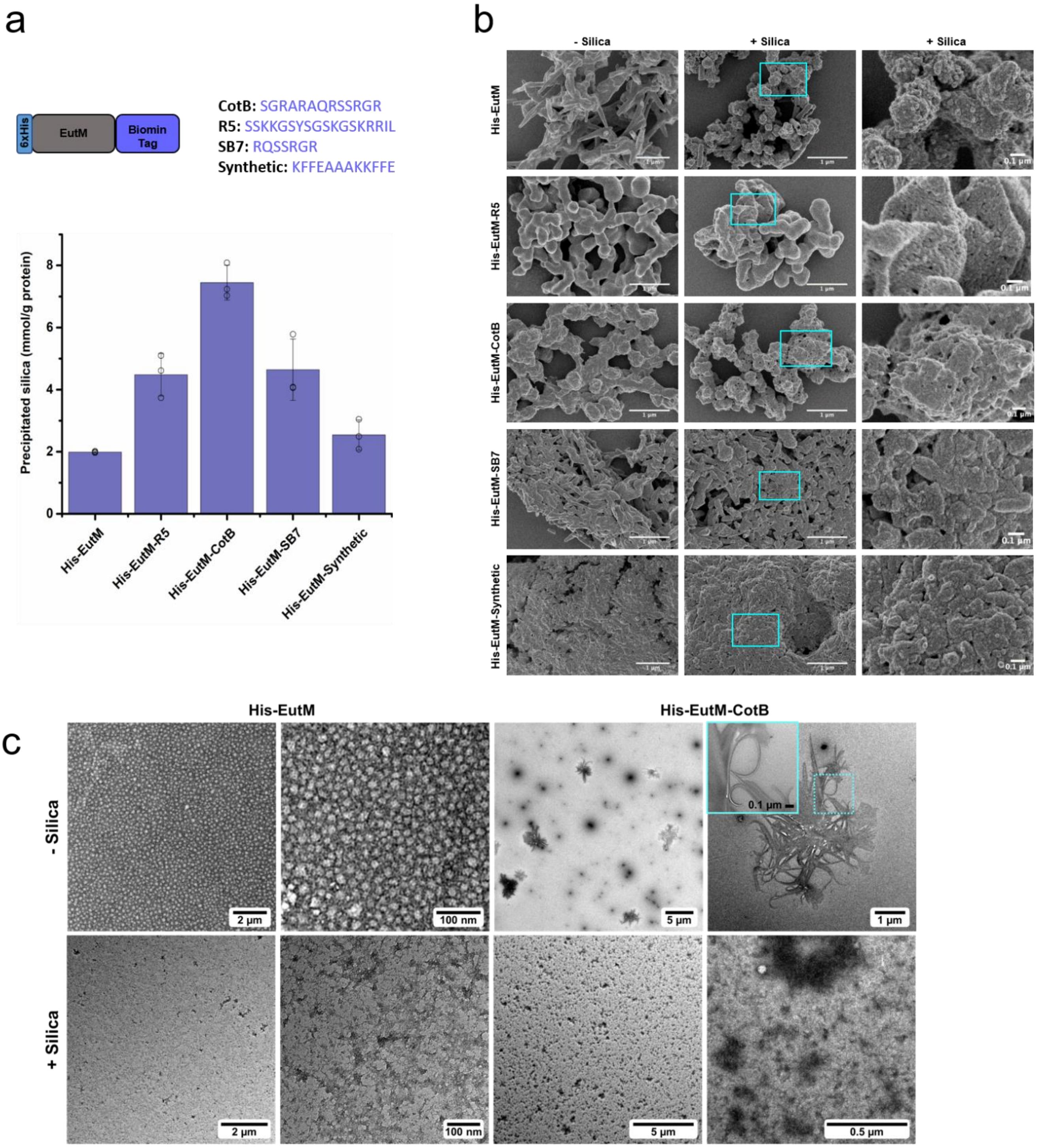
a
6xHis
EutM
Biomin Tag
CotB: SGRARAQRSSRGR
R5: SSKKGSYSGSKGSKRRIL
SB7: RQSSRGR
Synthetic: KFFEAAAKKFFE
Precipitated silica (mmol/g protein)
His-EutM
His-EutM-R5
His-EutM-CotB
His-EutM-SB7
His-EutM-Synthetic
b
- Silica
+ Silica
+ Silica
His-EutM
His-EutM-R5
His-EutM-CotB
His-EutM-SB7
His-EutM-Synthetic
c
His-EutM
His-EutM-CotB
- Silica
+ Silica
2 µm
100 nm
5 µm
1 µm
0.1 µm
0.5 µm

Supplementary Fig. 10. Characterization of silica binding and precipitation by EutM biomineralization peptide fusion proteins.

a Four silica binding peptides were fused to His-EutM (top panel) and silica binding to the purified proteins (1 mg/mL protein incubated with 100 mM silica for 2 h) compared to His-EutM. Silica precipitation was quantified spectrophotometrically with the molybdate blue assay. Data are shown as mean values ± SD and error bars represent the standard deviations of three independent biological replicate cultures. Colored bars represent mean values and back symbols represent data points. Source data are provided as a Source Data file.

b The same protein scaffolds (1 mg/mL) incubated without (- silica; left column) and with (+ silica (100 mM); middle, and right) for 2 h were visualized by SEM. Deposition of silica increases surface roughness and porosity, and also forms silica nanoparticle aggregates on the surface. His-EutM-CotB shows the strongest silica precipitation. His-EutM also precipitates silica due to its His-tag and positive charge[4]. Representative images from one set of purified proteins are shown.

c TEM grids were coated with His-EutM and His-EutM-CotB scaffolds (0.1 mg/mL purified protein solutions) and incubated without (-silica) and with (+ silica, 100 mM) for 1 h and visualized by TEM. His-EutM formed uniform structures, repeated at a fixed length scale. A relative homogenous layer of silica was deposited on the His-EutM layer. His-EutM-CotB did not form organized structures with a specific length-scale, but instead formed curved, fibrillar structures. These structures though promoted the deposition of silica that appears to be more granular and with larger particles compared to His-EutM. Representative images from one set of purified proteins are shown.

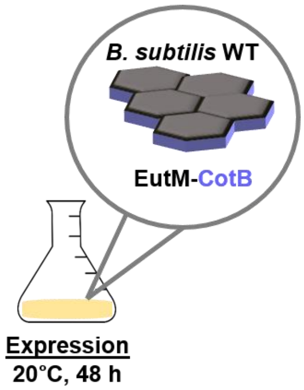

	Silica concentration (mM)					
Curing Time	50	100	200	300	400	500
10 min	Liquid	Liquid	Liquid	Hard solid	Hard solid	Hard solid
1 h	Liquid	Very soft gel	Very soft gel	Hard solid	Hard solid	Hard solid
2 h	Liquid	Very soft gel	Soft gel	Hard solid	Hard solid	Hard solid
24 h	Minor gelation	Soft gel (Control)<br>Soft Solid (EutMCotB)	Hard solid	Hard solid	Hard solid	Hard solid

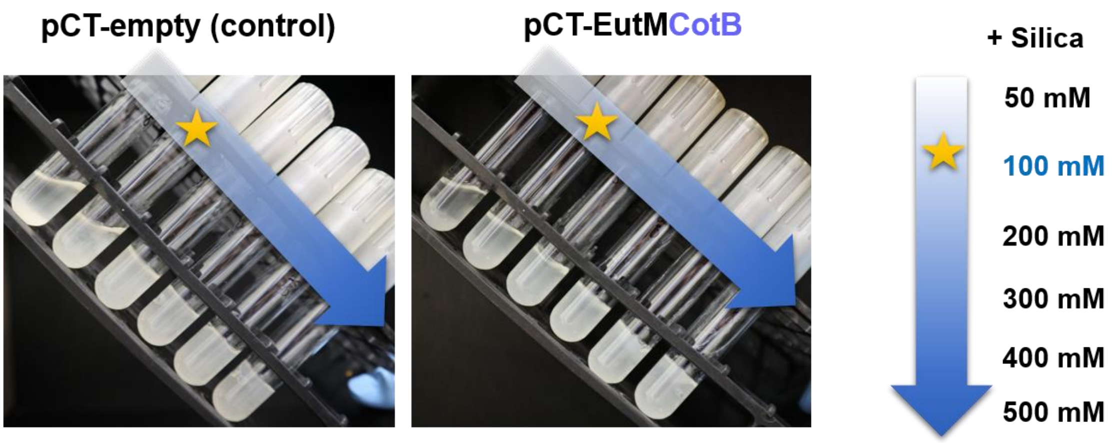

Supplementary Fig. 11. Comparison and optimization of silica gel formation by *B. subtilis* WT cultures expressing EutM-CotB scaffolds or no scaffolds.

B. subtilis WT pCT-empty (control) or pCT-EutMCotB cultures were grown in SMM at 20°C for 48 h after induction of protein expression. Different silica concentrations (50-500 mM) were then added to 2.5 mL culture samples and silica gel formation followed for 24 h at 20°C. The addition of 100 mM silica created a soft silica gel after 24 h with EutM-CotB expressing culture samples while control culture samples did not yield a solid gel. Lower silica concentration did not form a gel and higher concentrations rapidly formed a hard solid, regardless of culture sample (top panel). Bottom panel shows the solidification of these samples with increasing silica concentrations added (left to right). Cultures expressing EutM-CotB solidified with 100 mM silica (labeled with a star) while the control culture sample remained liquid with 100 mM silica.

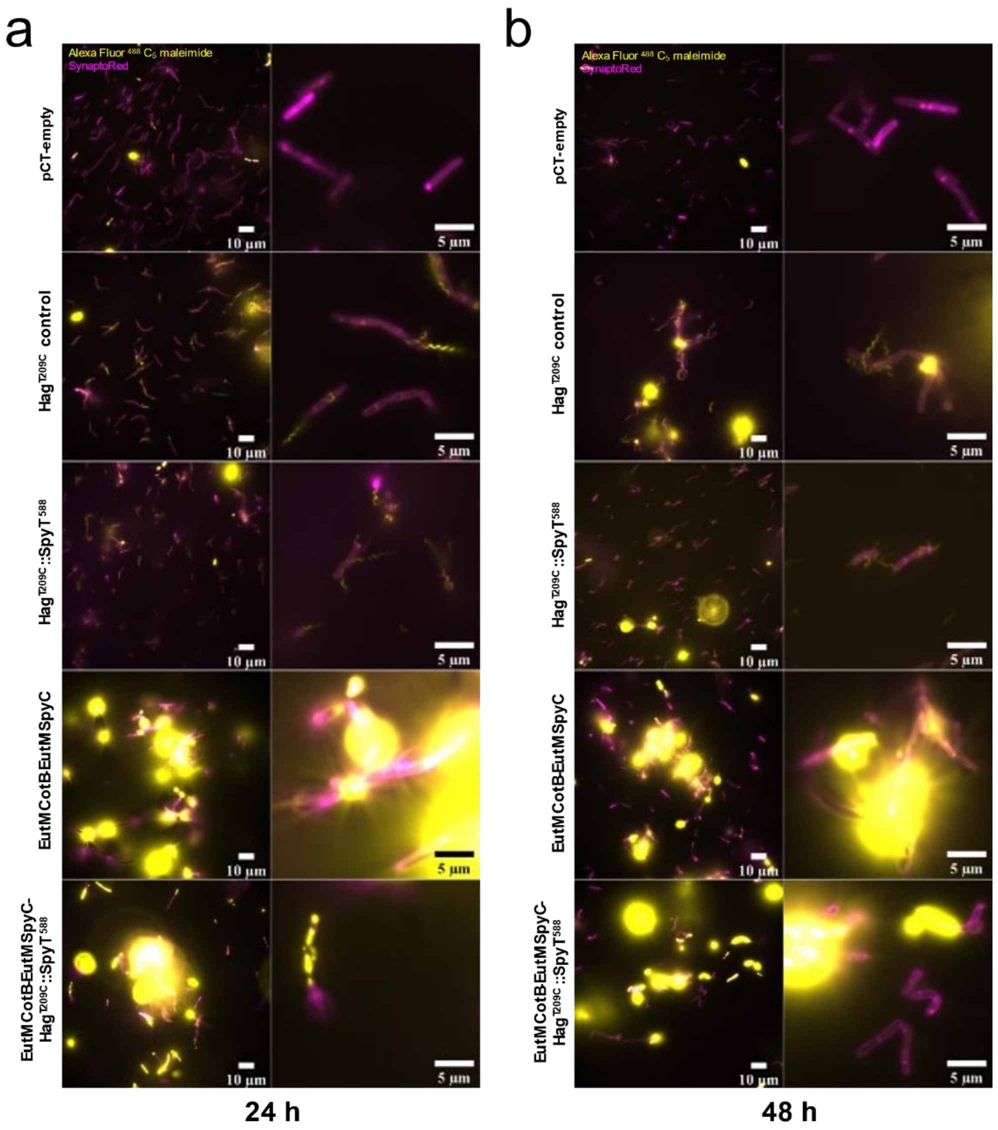

Supplementary Fig. 12. Visualization of cell phenotypes of *B. subtilis* Δ*lyt*C Δ*flh*G cultures transformed with different plasmids.

B. subtilis Δ*lyt*C Δ*flh*G was transformed with plasmids for the expression of modified flagella (pRBBm34-Hag^{T209C} and pRBBm34-Hag^{T209C}::SpyT^{588}), EutM scaffold building blocks (pCT-EutMCotB-EutMSpyC and pCT-empty(control)), and SpyTagged flagella and EutM scaffold building blocks (pCT-EutMCotB-EutMSpyC-Hag^{T209C}::SpyT^{588}). Strains harboring the pCT plasmids were grown for up to 48 h in SMM at 20°C under induction condition to express EutM scaffolds. Strains harboring the pRBBm34 plasmids were grown under the same conditions, but without induction. After 24 (**a**) and 48 h (**b**), samples were stained with the cysteine reactive dye Alexa Fluor^{TM} 488 C_5 maleimide and the cell membrane dye SynaptoRed^{TM} C2. Samples from EutM scaffold building block expressing cultures contained secreted scaffolds that appeared to be stained with the cysteine reactive Alexa Fluor dye, presumably due to labeling of one or both Cys residues present in the EutM protein. (Images generated for each strain in **a** and **b** are representative of cultures selected from three biological replicate cultures.)

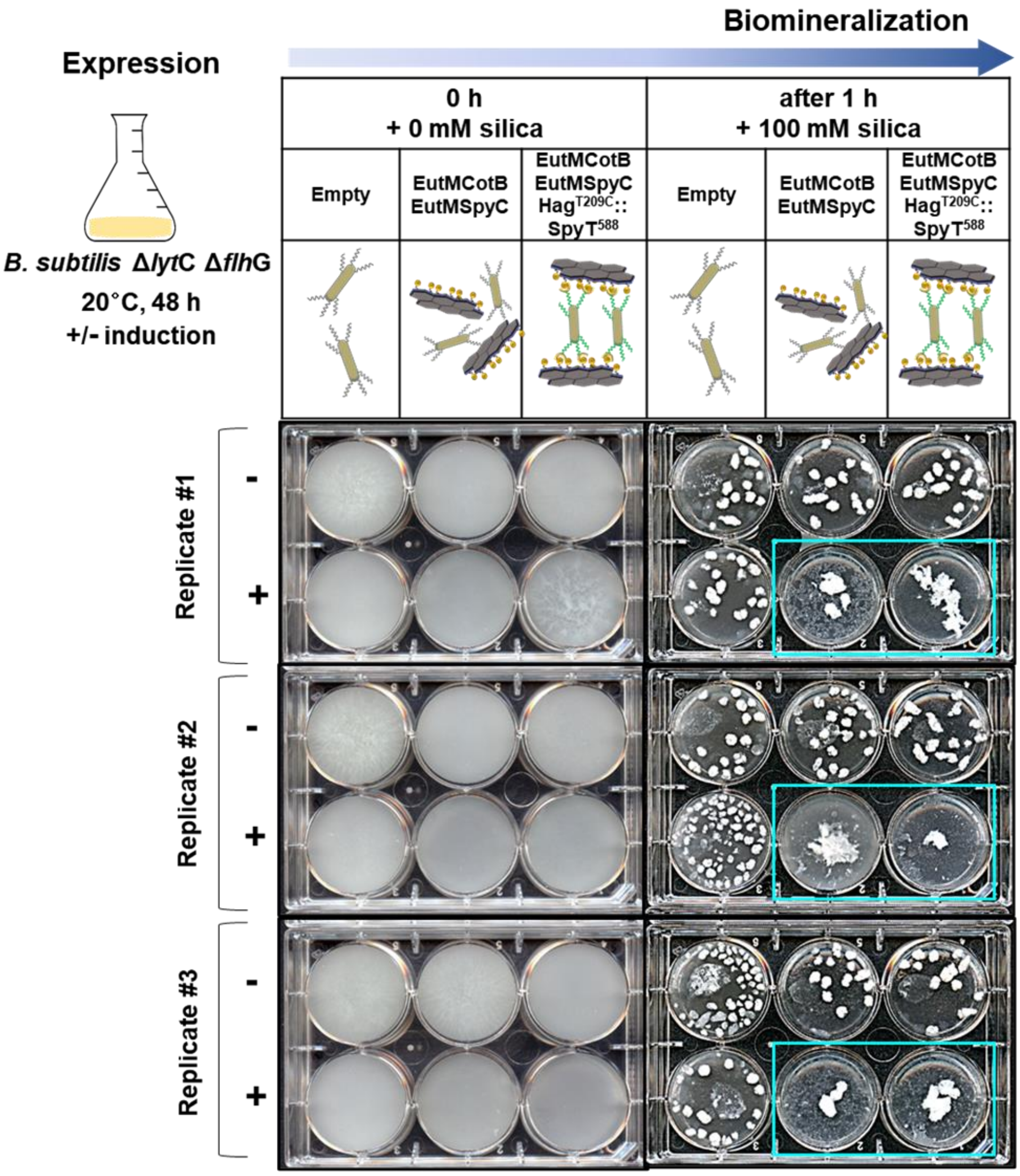

Supplementary Fig. 13. Silica biomineralization by engineered *B. subtilis* Δ*lyt*C Δ*flh*G cultures.

B. subtilis Δ*lytC* Δ*flhG* strains harboring three different pCT plasmids (second row) were grown in SMM at 20°C for 48 h under uninduced (-) and induced (+) conditions for protein expression. 5 mL cultures were then transferred to 6-well plates for silica biomineralization with 100 mM silica at 20°C, 100 rpm for 1 h. Experiments were performed with three biological culture replicates that are shown. Only cultures that co-expressed EutM-CotB and EutM-SpyCatcher formed aggregated silica materials upon induction. The aggregation appears to be more pronounced in cultures that also co-expressed SpyTag labeled flagella for cross-linking of cells and EutM scaffolds.

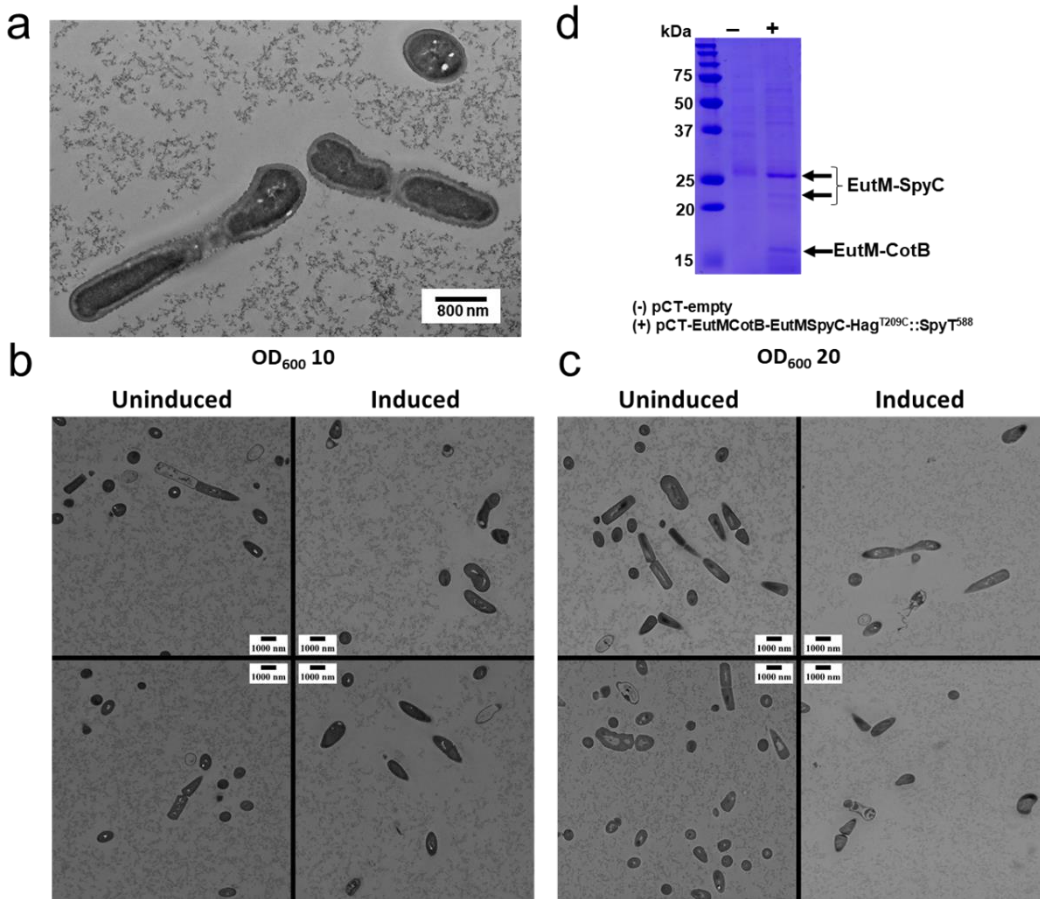

Supplementary Fig. 14. Additional characterization of *Bacillus* silica biocomposites.

a Close-up TEM image of cells with surrounding clear zone from thin sections of silica blocks formed with induced cultures grown for 48 h and incubated with 200 mM silica shown in **Fig. 7b**.

b, c Silica blocks generated from concentrated cultures with cell densities adjusted to OD_{600} of 10 or 20 were viewed by TEM for comparison to silica gel blocks directly formed from cultures (**Fig. 7**). *B. subtilis ΔlytC ΔflhG* pCT-EutMCotB-EutMSpyC-Hag^{T209C}::$SpyT^{588}$ was first grown under inducing (induced) or non-inducing (uninduced) conditions for protein expression (SMM, 20°C, 48 h). Cultures were centrifuged to collect secreted scaffolds and cells and resuspended in collected SMM supernatants to OD 10 (**b**) or 20 (**c**). Silica gel blocks were then formed by adding 200 mM silica and 1 mL aliquots of the cultures were transferred to syringes as molds for curing at 25°C. After 5 h of curing at 25°C, solid gel plugs were cut into 2 mm^3 pieces and prepared for thin sectioning. Cells were observed in clusters for both the uninduced and induced cultures, however only the induced culture contained clear zones around cells as was observed with unconcentrated cultures (**Fig. 7**).

d Silica gel blocks from 1 mL cultures (see **Fig. 6b**) were dissolved in SDS-PAGE loading buffer to confirm the presence of EutM scaffold proteins in the silica blocks by SDS-PAGE. Silica blocks made from empty vector control (-) cultures show no scaffold proteins while silica blocks from the EutMCotB-EutMSpyC-Hag^{T209C}::$SpyT^{588}$ induced (+) cultures have bands with the expected sizes for EutM-SpyCatcher and EutM-CotB.

Images and data are representative of silica blocks generated from two independent cultures. Source data are provided as a Source Data file.

Supplementary Table 1. Amino acid sequences of signal peptides.

Signal peptides	Amino acid sequence	Source	Reference
SacB	MNIKKFAKQATVLTFTTALLAGGATQAFAAST	*B. subtilis*	[Gilbert, 2017][1]
XynA	MFKFKKNFLVGLSAALMSISLFSATASAAST	*B. subtilis*	[Gilbert, 2017][1]
YngK	MYIKKCIGSILFLLLFCSSALPAKAAST	*B. megaterium*	[Stammen, 2010][5]
CelA	MKRSISIFITCLLITLLTMGGMIASPASAAST	*B. subtilis*	[Gilbert, 2017][1]
LipA	MKKVLMAFIICLSLILSVLAAPPSGAKAAST	*B. megaterium*	[Stammen, 2010][5]

Supplementary Table 2. Storage modulus (G') and loss modulus (G'') measurements of silica gels created with *B. subtilis* strains transformed with different plasmids. Shown are the means and standard deviations of angular frequency data from three silica blocks made from three different cultures (biological replicates) for each strain.

	**Storage modulus (G')**		
**Angular frequency (rad/S)**	**pCT-empty**	**pCT-EutMCotB-EutMSpyC**	**pCT-EutMCotB-EutMSpyC-Hag^{T209C}::SpyT^{588}**
100	1.05.E-03 ± 2.49.E-04	1.29.E-03 ± 7.29.E-05	1.36.E-03 ± 2.45.E-04
63.0957	1.01.E-03 ± 2.63.E-04	1.21.E-03 ± 7.88.E-05	1.36.E-03 ± 2.90.E-04
39.8107	9.60.E-04 ± 2.52.E-04	1.18.E-03 ± 5.22.E-05	1.31.E-03 ± 2.45.E-04
25.1189	9.38.E-04 ± 2.40.E-04	1.14.E-03 ± 4.60.E-05	1.27.E-03 ± 2.16.E-04
15.8489	9.06.E-04 ± 2.29.E-04	1.10.E-03 ± 4.87.E-05	1.24.E-03 ± 2.04.E-04
10	8.84.E-04 ± 2.19.E-04	1.07.E-03 ± 4.28.E-05	1.20.E-03 ± 1.77.E-04
6.30957	8.59.E-04 ± 2.07.E-04	1.02.E-03 ± 5.88.E-05	1.16.E-03 ± 1.52.E-04
3.98107	8.34.E-04 ± 1.59.E-04	1.01.E-03 ± 5.39.E-05	1.13.E-03 ± 1.19.E-04
2.51189	8.32.E-04 ± 1.82.E-04	9.70.E-04 ± 5.13.E-05	1.11.E-03 ± 1.31.E-04
1.58489	8.13.E-04 ± 1.57.E-04	9.11.E-04 ± 4.23.E-05	1.06.E-03 ± 1.10.E-04
1	7.82.E-04 ± 1.36.E-04	8.92.E-04 ± 5.50.E-05	1.06.E-03 ± 1.13.E-04
	**Loss modulus (G'')**		
**Angular frequency (rad/S)**	**pCT-empty**	**pCT-EutMCotB-EutMSpyC**	**pCT-EutMCotB-EutMSpyC-Hag^{T209C}::SpyT^{588}**
100	1.07.E-04 ± 2.81.E-05	9.03.E-05 ± 5.69.E-05	4.37.E-05 ± 1.23.E-04
63.0957	1.04.E-04 ± 6.01.E-05	1.12.E-04 ± 2.32.E-05	7.73.E-05 ± 3.30.E-05
39.8107	1.02.E-04 ± 4.18.E-05	9.05.E-05 ± 7.49.E-06	7.16.E-05 ± 1.50.E-05
25.1189	8.00.E-05 ± 2.92.E-05	9.48.E-05 ± 2.08.E-05	7.30.E-05 ± 1.52.E-05
15.8489	8.52.E-05 ± 3.16.E-05	9.18.E-05 ± 2.48.E-05	6.45.E-05 ± 2.34.E-05
10	8.24.E-05 ± 2.74.E-05	9.03.E-05 ± 1.17.E-05	7.18.E-05 ± 3.58.E-05
6.30957	9.71.E-05 ± 3.07.E-05	9.46.E-05 ± 2.48.E-05	8.12.E-05 ± 2.96.E-05
3.98107	9.86.E-05 ± 2.54.E-05	7.06.E-05 ± 3.59.E-05	7.15.E-05 ± 2.97.E-05
2.51189	9.03.E-05 ± 1.29.E-05	9.40.E-05 ± 5.46.E-05	9.47.E-05 ± 2.95.E-05
1.58489	8.31.E-05 ± 2.09.E-05	1.40.E-04 ± 3.93.E-05	9.31.E-05 ± 1.07.E-05
1	1.27.E-04 ± 2.82.E-05	1.34.E-04 ± 3.71.E-05	1.09.E-04 ± 2.25.E-05

Supplementary Table 3. Statistical analysis of rheology data.

T-test and one-way analysis of variance (ANOVA) were performed on G’ (storage modulus) and G” (loss modulus) angular frequency averages from **Supplementary Table 2**. T-Test analysis was performed to determine the statistical significance of rheology data between two groups of angular frequency data (G’ or G”) from *B. subtilis* strains transformed with the shown plasmids. ANOVA analysis was performed on three groups of angular frequency data (G’ or G”) from *B. subtilis* strains. All *p*-values are less than 0.05, except for the G” groups of pCT-empty and pCT-EutMCotB-EutMSpyC.

**T-test (Paired Two sample for Means)**	
**Statistical analysis of storage modulus (G’) between two groups**	***P*-value**
pCT-empty vs. pCT-EutMCotB-EutMSpyC	1.29E-07
pCT-empty vs. pCT-EutMCotB-EutMSpyC-Hag^{T209C}::SpyT^{588}	3.19E-11
pCT-EutMCotB-EutMSpyC vs. pCT-EutMCotB-EutMSpyC-Hag^{T209C}::SpyT^{588}	4.79E-09
**Statistical analysis of loss modulus (G’’) between two groups**	***P*-value**
pCT-empty vs. pCT-EutMCotB-EutMSpyC	0.54
pCT-empty vs. pCT-EutMCotB-EutMSpyC-Hag^{T209C}::SpyT^{588}	0.01
pCT-EutMCotB-EutMSpyC vs. pCT-EutMCotB-EutMSpyC-Hag^{T209C}::SpyT^{588}	7.86E-04
**ANOVA (Single-factor, One-way)**	
**Statistical analysis of storage modulus (G’) between three groups**	***P*-value**
pCT-empty vs. pCT-EutMCotB-EutMSpyC vs. pCT-EutMCotB-EutMSpyC-Hag^{T209C}::SpyT^{588}	1.05E-06
**Statistical analysis of loss modulus (G’’) between three groups**	***P*-value**
pCT-empty vs. pCT-EutMCotB-EutMSpyC vs. pCT-EutMCotB-EutMSpyC-Hag^{T209C}::SpyT^{588}	0.01

Supplementary references

1 Gilbert, C., Howarth, M., Harwood, C. R. & Ellis, T. Extracellular self-assembly of functional and tunable protein conjugates from *Bacillus subtilis*. *ACS Synth Biol* 6, 957-967 (2017).

2 Schmidt-Dannert, S., Zhang, G., Johnston, T., Quin, M. B. & Schmidt-Dannert, C. Building a toolbox of protein scaffolds for future immobilization of biocatalysts. *Appl Microbiol Biotechnol* 102, 8373-8388 (2018).

3 Zhang, G., Schmidt-Dannert, S., Quin, M. B. & Schmidt-Dannert, C. Protein-based scaffolds for enzyme immobilization. *Methods Enzymol* 617, 323-362 (2019).

4 Shimizu, K. *et al.* Glassin, a histidine-rich protein from the siliceous skeletal system of the marine sponge *Euplectella*, directs silica polycondensation. *Proc Natl Acad Sci USA* 112, 11449-11454 (2015).

5 Stammen, S. *et al.* High-yield intra- and extracellular protein production using *Bacillus megaterium*. *Appl Environ Microbiol* 76, 4037-4046 (2010).
