# Supplementary material for: Engineering Bacillus subtilis for the formation of a durable living biocomposite material
Source: Nat Commun. 2021 Dec 8;12:7133. doi: 10.1038/s41467-021-27467-2 (PMC8654922; doi:10.1038/s41467-021-27467-2)
Supplement: Supplementary file 3 — Description of Additional Supplementary Files [file 41467_2021_27467_MOESM3_ESM.pdf]

Description of Additional Supplementary Files

File Name: Supplementary Data 1
Description: Plasmids and strains used in this study.

File Name: Supplementary Data 2
Description: Amino acid sequences of proteins and peptides in this study.

File Name: Supplementary Data 3
Description: Nucleotide sequences of proteins and peptides used in this study.

File Name: Supplementary Data 4
Description: List of primers used in this study.
