# Supplementary material for: Engineering Bacillus subtilis for the formation of a durable living biocomposite material
Source: Nat Commun. 2021 Dec 8;12:7133. doi: 10.1038/s41467-021-27467-2 (PMC8654922; doi:10.1038/s41467-021-27467-2)
Supplement: Supplementary file 8 — Reporting Summary [file 41467_2021_27467_MOESM8_ESM.pdf]

nature portfolio

Corresponding author(s): Prof. Claudia Schmidt-Dannert

Last updated by author(s): November 10 2021

Reporting Summary

Nature Portfolio wishes to improve the reproducibility of the work that we publish. This form provides structure for consistency and transparency in reporting. For further information on Nature Portfolio policies, see our Editorial Policies and the Editorial Policy Checklist.

Statistics

For all statistical analyses, confirm that the following items are present in the figure legend, table legend, main text, or Methods section.

n/a	Confirmed	
☐	☒	The exact sample size (n) for each experimental group/condition, given as a discrete number and unit of measurement
☐	☒	A statement on whether measurements were taken from distinct samples or whether the same sample was measured repeatedly
☐	☒	The statistical test(s) used AND whether they are one- or two-sided<br>*Only common tests should be described solely by name; describe more complex techniques in the Methods section.*
☒	☐	A description of all covariates tested
☒	☐	A description of any assumptions or corrections, such as tests of normality and adjustment for multiple comparisons
☐	☒	A full description of the statistical parameters including central tendency (e.g. means) or other basic estimates (e.g. regression coefficient) AND variation (e.g. standard deviation) or associated estimates of uncertainty (e.g. confidence intervals)
☐	☒	For null hypothesis testing, the test statistic (e.g. F, t, r) with confidence intervals, effect sizes, degrees of freedom and P value noted<br>*Give P values as exact values whenever suitable.*
☒	☐	For Bayesian analysis, information on the choice of priors and Markov chain Monte Carlo settings
☒	☐	For hierarchical and complex designs, identification of the appropriate level for tests and full reporting of outcomes
☒	☐	Estimates of effect sizes (e.g. Cohen's d, Pearson's r), indicating how they were calculated

Our web collection on statistics for biologists contains articles on many of the points above.

Software and code

Policy information about availability of computer code

Data collection	LC-MS data was collected using Thermo Scientific LTQ Orbitrap Velos mass spectrometer and the data were analyzed and viewed using the PEAKS Xpro Studio 10.6 software (Bioinformatics Solutions Inc., Waterloo, ON, Canada).<br>Rheology data was collected using DMA Rheometer (TA Instruments RSA-G2).<br>Flagella and Spore staining data was captured on Nikon Eclipse 90i microscope with a 100X 1.3 numerical aperture oil-immersion objective with a Nikon D2-Fi2 color camera running on Nikon Elements software.<br>SEM images were captured from Hitachi SU8230 field emission gun scanning electron microscope.<br>TEM samples were examined using a JEOL-JEM-1400Plus transmission electron microscope with a LaB6 tungsten filament at 60 kV and the images were acquired using an Advanced Microscopy Techniques XR16 camera using AMT capture Engine software version 7.0.0.187.<br>DIC images were captured using a Hamamatsu Orca Flash 4.0 v2 CMOS monochrome camera in black and white using Nikon NIS-Elements software ver 5.2.1.00. Images were cropped, scale bars added, and channels were colorized and merged using Fiji v. 1.53.<br>Flagella RYU staining images were converted to grayscale and had the contrast and brightness increased by 10 using GNU Image Manipulation Program (GIMP) v. 2.8.18 (https://www.gimp.org/).
Data analysis	Statistical analysis for OD and pH data was performed using Origin v. 9.1. Statistical analysis for rheology data was performed using Microsoft Excel 2016.

For manuscripts utilizing custom algorithms or software that are central to the research but not yet described in published literature, software must be made available to editors and reviewers. We strongly encourage code deposition in a community repository (e.g. GitHub). See the Nature Portfolio guidelines for submitting code & software for further information.

Data

Policy information about availability of data

All manuscripts must include a data availability statement. This statement should provide the following information, where applicable:

- Accession codes, unique identifiers, or web links for publicly available datasets
- A description of any restrictions on data availability
- For clinical datasets or third party data, please ensure that the statement adheres to our policy

All data generated or analyzed in this study are included in this article and accompanying Supplementary Information, Supplementary Tables and Source Data File. E. coli B. subtilis 168 strains used are commercially available. Engineered Bacillus strains and plasmids created in this study can be made available subject to an MTA that can be requested by contacting the corresponding author. Protein Data Bank data sets (PDB: 5WJX [http://dx.doi.org/10.2210/pdb5wjx/pdb], PDB: 6GOW [http://dx.doi.org/10.2210/pdb6gow/pdb], PDB: 4MLI [http://dx.doi.org/10.2210/pdb4mli/pdb]) were used to generate protein structure representations.

Field-specific reporting

Please select the one below that is the best fit for your research. If you are not sure, read the appropriate sections before making your selection.

☒ Life sciences ☐ Behavioural & social sciences ☐ Ecological, evolutionary & environmental sciences

For a reference copy of the document with all sections, see nature.com/documents/nr-reporting-summary-flat.pdf

Life sciences study design

All studies must disclose on these points even when the disclosure is negative.

Sample size	Required experimental sample sizes were based on common practice of taking measurements from three independent biological replicates to determine spread of data. For OD600 and pH data, the statistical analysis for n=3 sample size was use to determ mean +/- SD as indicated by error bars in the shown plots. For rheology experiments, data from three independent biological replicates were also used to determine statistical significance using paired T-test and one-way ANOVA analysis in addition to representation of mean +/- SD as using error bars.
Data exclusions	No data were excluded from analysis.
Replication	All measurements were replicated successfully at least three times. In addition, final experiments were preceded by multiple pilot experiments. Independent biological triplicates were used for all applicable experiments.
Randomization	No sample allocation method was used. Randomization is not applicable for cell density, pH or material characterization data in this study.
Blinding	No blinding was applied for studies performed as no other groups were participated.

Reporting for specific materials, systems and methods

We require information from authors about some types of materials, experimental systems and methods used in many studies. Here, indicate whether each material, system or method listed is relevant to your study. If you are not sure if a list item applies to your research, read the appropriate section before selecting a response.

Materials & experimental systems

n/a	Involved in the study	
☒	☐	Antibodies
☒	☐	Eukaryotic cell lines
☒	☐	Palaeontology and archaeology
☒	☐	Animals and other organisms
☒	☐	Human research participants
☒	☐	Clinical data
☒	☐	Dual use research of concern

Methods

n/a	Involved in the study	
☒	☐	ChIP-seq
☒	☐	Flow cytometry
☒	☐	MRI-based neuroimaging
